# Machine learning models for abstract screening task - A systematic literature review application for health economics and outcome research

**DOI:** 10.1186/s12874-024-02224-3

**Published:** 2024-05-09

**Authors:** Jingcheng Du, Ekin Soysal, Dong Wang, Long He, Bin Lin, Jingqi Wang, Frank J. Manion, Yeran Li, Elise Wu, Lixia Yao

**Affiliations:** 1 Intelligent Medical Objects, Houston, TX USA; 2 Merck & Co., Inc, Rahway, NJ USA; 3 McWilliams School of Biomedical Informatics, University of Texas Health Science Center at Houston, Houston, TX, USA

**Keywords:** Machine learning, Deep learning, Text classification, Article screening, Systematic literature review

## Abstract

**Objective:**

Systematic literature reviews (SLRs) are critical for life-science research. However, the manual selection and retrieval of relevant publications can be a time-consuming process. This study aims to (1) develop two disease-specific annotated corpora, one for human papillomavirus (HPV) associated diseases and the other for pneumococcal-associated pediatric diseases (PAPD), and (2) optimize machine- and deep-learning models to facilitate automation of the SLR abstract screening.

**Methods:**

This study constructed two disease-specific SLR screening corpora for HPV and PAPD, which contained citation metadata and corresponding abstracts. Performance was evaluated using precision, recall, accuracy, and F1-score of multiple combinations of machine- and deep-learning algorithms and features such as keywords and MeSH terms.

**Results and conclusions:**

The HPV corpus contained 1697 entries, with 538 relevant and 1159 irrelevant articles. The PAPD corpus included 2865 entries, with 711 relevant and 2154 irrelevant articles. Adding additional features beyond title and abstract improved the performance (measured in Accuracy) of machine learning models by 3% for HPV corpus and 2% for PAPD corpus. Transformer-based deep learning models that consistently outperformed conventional machine learning algorithms, highlighting the strength of domain-specific pre-trained language models for SLR abstract screening. This study provides a foundation for the development of more intelligent SLR systems.

## Introduction

Systematic literature reviews (SLRs) are an essential tool in many areas of health sciences, enabling researchers to understand the current knowledge around a topic and identify future research and development directions. In the field of health economics and outcomes research (HEOR), SLRs play a crucial role in synthesizing evidence around unmet medical needs, comparing treatment options, and preparing the design and execution of future real-world evidence studies. SLRs provide a comprehensive and transparent analysis of available evidence, allowing researchers to make informed decisions and improve patient outcomes.

Conducting a SLR involves synthesizing high-quality evidence from biomedical literature in a transparent and reproducible manner, and seeks to include all available evidence on a given research question, and provides some assessment regarding quality of the evidence [1, 2]. To conduct an SLR one or more bibliographic databases are queried based on a given research question and a corresponding set of inclusion and exclusion criteria, resulting in the selection of a relevant set of abstracts. The abstracts are reviewed, further refining the set of articles that are used to address the research question. Finally, appropriate data is systematically extracted from the articles and summarized [1, 3]. 

The current approach to conducting a SLR is through manual review, with data collection, and summary done by domain experts against pre-specified eligibility criteria. This is time-consuming, labor-intensive, expensive, and non-scalable given the current more-than linear growth of the biomedical literature [4]. Michelson and Reuter estimate that each SLR costs approximately $141,194.80 and that on average major pharmaceutical companies conduct 23.36 SLRs, and major academic centers 177.32 SLRs per year, though the cost may vary based on the scope of different reviews [4]. Clearly automated methods are needed, both from a cost/time savings perspective, and for the ability to effectively scan and identify increasing amounts of literature, thereby allowing the domain experts to spend more time analyzing the data and gleaning the insights.

One major task of SLR project that involves large amounts of manual effort, is the abstract screening task. For this task, selection criteria are developed and the citation metadata and abstract for articles tentatively meeting these criteria are retrieved from one or more bibliographic databases (e.g., PubMed). The abstracts are then examined in more detail to determine if they are relevant to the research question(s) and should be included or excluded from further consideration. Consequently, the task of determining whether articles are relevant or not based on their titles, abstracts and metadata can be treated as a binary classification task, which can be addressed by natural language processing (NLP). NLP involves recognizing entities and relationships expressed in text and leverages machine-learning (ML) and deep-learning (DL) algorithms together with computational semantics to extract information. The past decade has witnessed significant advances in these areas for biomedical literature mining. A comprehensive review on how NLP techniques in particular are being applied for automatic mining and knowledge extraction from biomedical literature can be found in Zhao et al. [5].

## Materials and methods

The aims of this study were to: (1) identify and develop two disease-specific corpora, one for human papillomavirus (HPV) associated diseases and the other for pneumococcal-associated pediatric diseases suitable for training the ML and DL models underlying the necessary NLP functions; (2) investigate and optimize the performance of the ML and DL models using different sets of features (e.g., keywords, Medical Subject Heading (MeSH) terms [6]) to facilitate automation of the abstract screening tasks necessary to construct a SLR. Note that these screening corpora can be used as training data to build different NLP models. We intend to freely share these two corpora with the entire scientific community so they can serve as benchmark corpora for future NLP model development in this area.

### SLR corpora preparation

Two completed disease-specific SLR studies by Merck & Co., Inc., Rahway, NJ, USA were used as the basis to construct corpora for abstract-level screening. The two SLR studies were both relevant to health economics and outcome research, including one for human papillomavirus (HPV) associated diseases (referred to as the *HPV* corpus), and one for pneumococcal-associated pediatric diseases (which we refer to as the *PAPD* corpus). Both of the original SLR studies contained literature from PubMed/MEDLINE and EMBASE. Since we intended for the screening corpora to be released to the community, we only kept citations found from PubMed/MEDLINE in the finalized corpora. Because the original SLR studies did not contain the PubMed ID (PMID) for each article, we matched each article’s citation information (if available) against PubMed and then collected meta-data such as authors, journals, keywords, MeSH terms, publication types, etc., using PubMed Entrez Programming Utilities (E-utilities) Application Programming Interface (API). The detailed description of the two corpora can be seen in Table 1. Both of the resulting corpora are publicly available at [https://github.com/Merck/NLP-SLR-corpora ].

**Table 1:** Descriptions of SLR abstract-level screening corpora

		HPV corpus	PAPD corpus
Study aim		To identify the available peer-reviewed evidence on the prevalence of HPV detected in head and neck squamous cell carcinomas (HNSCCs)	To gain an understanding of the burden of Pneumococcal Disease for pediatric patients (humanistic, economic, and epidemiological) through the development of a systematic literature review
Study period		2012 to 2020	2016 to 2021
Inclusion criteria		● adults (age > = 13) with histologically confirmed invasive HNSCCs (oral cavity, oropharynx, larynx, hypopharynx) ● report type-specific HPV DNA prevalence (2012–2014) or any HPV DNA prevalence (2015–2020)	● Pediatric patients (0–18 years old) with pneumococcal disease ● Clinical manifestations of Pneumococcal Disease including: o Pneumonia (including community acquired, hospital acquired and ventilator acquired pneumonia, and non-bacteremic pneumococcal pneumonia [NBPP]) o Meningitis (including post-meningitis sequalae) o Acute otitis media o Bacteremia (sepsis, septicemia) o Empyema • High risk population subgroups, including: o Cancer o Immunocompromised o HIV o Renal disease o Asplenia o Diabetes o Heart conditions o Lung conditions o Sickle cell disease o Cochlear implants o Cerebrospinal fluid leaks
Exclusion criteria		● Children (age < 13 years) years only ● HPV-infected subjects only ● Immunocompromised populations only (e.g., HIV-infected) ● Specific high-risk populations only (e.g., smokers, people who abuse alcohol) ● Patients with specific, not targeted diseases or undergoing specific treatments only ● Other special populations (e.g., prison inmates, immigrants, ethnic minority populations only) ● Language not English ● Conference proceedings ● No abstract ● Narrative review (however, highly relevant reviews were included to be perused in full text for primary references) ● Small study (*N* < 25) ● Clinical guideline	● Mixed pediatric/adult populations without segregated results
Corpus statistics	Total citations	1697	2865
	Included citations	538	711
	Excluded citations	1159	2154

### Machine learning algorithms

Although deep learning algorithms have demonstrated superior performance on many NLP tasks, conventional machine learning algorithms have certain advantages, such as low computation costs and faster training and prediction speed.

We evaluated four traditional ML-based document classification algorithms, XGBoost [7], Support Vector Machines (SVM) [8], Logistic regression (LR) [9], and Random Forest [10] on the binary inclusion/exclusion classification task for abstract screening. Salient characteristics of these models are as follows:

1. XGBoost: Short for “eXtreme Gradient Boosting”, XGBoost is a boosting-based ensemble of algorithms that turn weak learners into strong learners by focusing on where the individual models went wrong. In Gradient Boosting, individual weak models train upon the difference between the prediction and the actual results [7]. We set max_depth at 3, n_estimators at 150 and learning rate at 0.7.
2. Support vector machine (SVM): SVM is one of the most robust prediction methods based on statistical learning frameworks. It aims to find a hyperplane in an N-dimensional space (where N = the number of features) that distinctly classifies the data points [8]. We set C at 100, gamma at 0.005 and kernel as radial basis function.
3. Logistic regression (LR): LR is a classic statistical model that in its basic form uses a logistic function to model a binary dependent variable [9]. We set C at 5 and penalty as l2.
4. Random forest (RF): RF is a machine learning technique that utilizes ensemble learning to combine many decision trees classifiers through bagging or bootstrap aggregating [10]. We set n_estimators at 100 and max_depth at 14.

These four algorithms were trained for both the *HPV* screening task and the *PAPD* screening task using the corresponding training corpus.

For each of the four algorithms, we examined performance using (1) only the baseline feature criteria (title and abstract of each article), and (2) with five additional meta-data features (MeSH, Authors, Keywords, Journal, Publication types.) retrieved from each article using the PubMed E-utilities API. Conventionally, title and abstract are the first information a human reviewer would depend on when making a judgment for inclusion or exclusion of an article. Consequently, we used title and abstract as the baseline features to classify whether an abstract should be included at the abstract screening stage. We further evaluated the performance with additional features that can be retrieved by PubMed E-utilities API, including MeSH terms, authors, journal, keywords and publication type. For baseline evaluation, we concatenated the titles and abstracts and extracted the TF-IDF (term frequency-inverse document frequency) vector for the corpus. TF-IDF evaluates how relevant a word is to a document in a collection of documents. For additional features, we extracted TF-IDF vector using each feature respectively and then concatenated the extracted vectors with title and abstract vector. XGBoost was selected for the feature evaluation process, due to its relatively quick computational running time and robust performance.

### Deep learning algorithms

Conventional ML methods rely heavily on manually designed features and suffer from the challenges of data sparsity and poor transportability when applied to new use cases. Deep learning (DL) is a set of machine learning algorithms based on deep neural networks that has advanced performance of text classification along with many other NLP tasks. Transformer-based deep learning models, such as BERT (Bidirectional encoder representations from transformers), have achieved state-of-the-art performance in many NLP tasks [11]. A *Transformer* is an emerging architecture of deep learning models designed to handle sequential input data such as natural language by adopting the mechanisms of attention to differentially weigh the significance of each part of the input data [12]. The BERT model and its variants (which use Transformer as a basic unit) leverage the power of transfer learning by first pre-training the models over 100’s of millions of parameters using large volumes of unlabeled textual data. The resulting model is then fine-tuned for a particular downstream NLP application, such as text classification, named entity recognition, relation extraction, etc. The following three BERT models were evaluated against both the *HPV* and *Pediatric pneumococcal* corpus using two sets of features (title and abstract versus adding all additional features into the text). For all BERT models, we used Adam optimizer with weight decay. We set learning rate at 1e-5, batch size at 8 and number of epochs at 20.

1. BERT base: this is the original BERT model released by Google. The BERT base model was pre-trained on textual data in the general domain, i.e., BooksCorpus (800 M words) and English Wikipedia (2500 M words) [11].
2. BioBERT base: as the biomedical language is different from general language, the BERT models trained on general textual data may not work well on biomedical NLP tasks. BioBERT was further pre-trained (based on original BERT models) in the large-scale biomedical corpora, including PubMed abstracts (4.5B words) and PubMed Central Full-text articles (13.5B words) [13].
3. PubMedBERT: PubMedBERT was pre-trained from scratch using abstracts from PubMed. This model has achieved state-of-the-art performance on several biomedical NLP tasks on Biomedical Language Understanding and Reasoning Benchmark [14].

### Text pre-processing and libraries that were used

We have removed special characters and common English words as a part of text pre-processing. Default tokenizer from scikit-learn was adopted for tokenization. Scikit-learn was also used for TF-IDF feature extraction and machine learning algorithms implementation. Transformers libraries from Hugging Face were used for deep learning algorithms implementation.

### Evaluation

Evaluation datasets were constructed from the *HPV* and *Pediatric pneumococcal* corpora and were split into training, validation and testing sets with a ratio of 8:1:1 for the two evaluation tasks: (1) ML algorithms performance assessment; and (2) DL algorithms performance assessment. Models were fitted on the training sets, and model hyperparameters were optimized on the validation sets and the performance were evaluated on the testing sets. The following major metrics are expressed by the noted calculations:

$$Precision = \frac{True positive}{True positive + False positive}$$

$$Recall = \frac{True positive}{True positive + False negative}$$

$$F1 score = \frac{2 \times Precision \times Recall}{Precision + Recall}$$

$$\begin{gathered} Accuracy = \\ \frac{{Truepositive + Truenegative}}{{Truepositive + Truenegative + Falsepositive + Falsenegative}} \\ \end{gathered}$$

Where *True positive* is an outcome where the model correctly predicts the positive (e.g., “included” in our tasks) class. Similarly, a *True negative* is an outcome where the model correctly predicts the negative class (e.g., “excluded” in our tasks). *False positive* is an outcome where the model incorrectly predicts the positive class, and a *False negative* is an outcome where the model incorrectly predicts the negative class. We have repeated all experiments five times and reported the mean scores with standard deviation.

## Results

Table 2 shows the baseline comparison using different feature combinations for the SLR text classification tasks using XGBoost. As noted, adding additional features in addition to title and abstract was effective in further improving the classification accuracy. Specifically, using all available features for the *HPV* classification increased accuracy by ?∼ 3% and F1 score by ?∼ 3%; using all available features for *Pediatric pneumococcal* classification increased accuracy by ?∼ 2% and F1 score by ?∼ 4%. As observed, adding additional features provided a stronger boost in precision, which contributed to the overall performance improvement.

**Table 2:** The features evaluation results using XGBoost as the classification algorithm. **Bold** indicates the best score

Task	Features	F1 score	Precision	Recall	Accuracy
*HPV*	Title + abstract	0.77(0.02)	0.69(0.02)	0.87(0.04)	0.83(0.01)
	Title + abstract + authors	0.77(0.02)	0.69(0.02)	0.87(0.04)	0.84(0.02)
	Title + abstract + keywords	0.77(0.01)	0.69(0.01)	0.88(0.02)	0.84(0.01)
	Title + abstract + journal	0.77(0.02)	0.69(0.02)	0.87(0.04)	0.83(0.01)
	Title + abstract + publication types	0.77(0.01)	0.69(0.02)	0.86(0.02)	0.84(0.01)
	Title + abstract + MeSH	**0.80(0.02)**	**0.72(0.03)**	0.89(0.03)	**0.86(0.02)**
	Title + abstract + authors + keywords + journal + MeSH + publication types	**0.80(0.02)**	**0.72(0.02)**	**0.90(0.03)**	**0.86(0.01)**
*PADA*	Title + abstract	0.74(0.02)	0.70(0.02)	0.79(0.04)	0.86(0.01)
	Title + abstract + authors	0.74(0.02)	0.70(0.02)	0.79(0.04)	0.86(0.01)
	Title + abstract + keywords	0.75(0.01)	0.71(0.01)	0.80(0.03)	0.87(0.01)
	Title + abstract + journal	0.74(0.02)	0.70(0.02)	0.79(0.04)	0.86(0.01)
	Title + abstract + publication types	0.74(0.02)	0.70(0.02)	0.79(0.04)	0.86(0.01)
	Title + abstract + MeSH	0.77(0.01)	**0.74(0.01)**	**0.81(0.02)**	**0.88(0.00)**
	Title + abstract + authors + keywords + journal + MeSH + publication types	**0.78(0.01)**	**0.74(0.01)**	**0.81(0.02)**	**0.88(0.00)**

The comparison of the article inclusion/exclusion classification task for four machine learning algorithms with all features is shown in Table 3. XGBoost achieved the highest accuracy and F-1 scores in both tasks. Table 4 shows the comparison between XGBoost and deep learning algorithms on the classification tasks for each disease. Both XGBoost and deep learning models consistently have achieved higher accuracy scores when using all features as input. Among all models, BioBERT has achieved the highest accuracy at 0.88, compared with XGBoost at 0.86. XGBoost has the highest F1 score at 0.8 and the highest recall score at 0.9 for inclusion prediction.

**Table 3:** The comparison among conventional machine learning algorithms using all features combination. **Bold** indicates the best score

Task	Algorithm	F1 score	Precision	Recall	Accuracy
*HPV*	XGBoost	**0.80(0.02)**	0.72(0.02)	**0.90(0.03)**	**0.86(0.01)**
	Support vector machine	0.71(0.01)	**0.75(0.01)**	0.67(0.02)	0.82(0.01)
	Logistics regression	0.74(0.01)	0.70(0.01)	0.78(0.02)	0.83(0.01)
	Random forest	0.75(0.03)	0.74(0.04)	0.76(0.03)	0.84(0.02)
*PADA*	XGBoost	**0.78(0.01)**	**0.74(0.01)**	**0.81(0.02)**	**0.88(0.00)**
	Support vector machine	0.74(0.01)	0.69(0.02)	0.80(0.02)	0.86(0.01)
	Logistics regression	0.73(0.00)	0.69(0.01)	0.78(0.02)	0.86(0.00)
	Random forest	0.69(0.01)	0.70(0.01)	0.69(0.03)	0.85(0.00)

**Table 4:** The comparison of machine learning and deep learning algorithms. **Bold** indicates the best score

Task	Algorithm	F1 score	Precision	Recall	Accuracy
*HPV*	XGBoost – title and abstract	0.77(0.02)	0.69(0.02)	0.87(0.04)	0.83(0.01)
	XGBoost – all features	**0.80(0.02)**	0.72(0.02)	**0.90(0.03)**	0.86(0.01)
	PubMedBERT – title and abstract	0.75(0.03)	0.72(0.11)	0.81(0.10)	0.83(0.05)
	PubMedBERT – all features	0.76(0.02)	0.77(0.02)	0.74(0.03)	0.87(0.01)
	BioBERT – title and abstract	0.74(0.01)	0.68(0.02)	0.81(0.05)	0.82(0.01)
	BioBERT – all features	0.76(0.03)	**0.86(0.04)**	0.68(0.03)	**0.88(0.01)**
	BERT base – title and abstract	0.68(0.03)	0.57(0.05)	0.86(0.04)	0.74(0.05)
	BERT base – all features	0.66(0.03)	0.62(0.12)	0.75(0.11)	0.78(0.06)
*PADA*	XGBoost – title and abstract	0.74(0.02)	0.70(0.02)	0.79(0.04)	0.86(0.01)
	XGBoost – all features	0.78(0.01)	0.74(0.01)	0.81(0.02)	0.88(0.00)
	PubMedBERT – title and abstract	0.79(0.02)	0.75(0.04)	**0.85(0.02)**	0.89(0.01)
	PubMedBERT – all features	**0.80(0.01)**	**0.79(0.03)**	0.80(0.01)	**0.90(0.01)**
	BioBERT – title and abstract	0.79(0.01)	0.74(0.03)	0.84(0.03)	0.89(0.01)
	BioBERT – all features	0.79(0.01)	0.76(0.02)	0.82(0.02)	0.89(0.01)
	BERT base – title and abstract	0.71(0.02)	0.62(0.03)	0.83 (0.01)	0.83(0.02)
	BERT base – all features	0.71(0.04)	0.65(0.06)	0.80 (0.03)	0.84(0.03)

## Discussions and conclusions

Abstract screening is a crucial step in conducting a systematic literature review (SLR), as it helps to identify relevant citations and reduces the effort required for full-text screening and data element extraction. However, screening thousands of abstracts can be a time-consuming and burdensome task for scientific reviewers. In this study, we systematically investigated the use of various machine learning and deep learning algorithms, using different sets of features, to automate abstract screening tasks. We evaluated these algorithms using disease-focused SLR corpora, including one for human papillomavirus (HPV) associated diseases and another for pneumococcal-associated pediatric diseases (PADA). The publicly available corpora used in this study can be used by the scientific community for advanced algorithm development and evaluation. Our findings suggest that machine learning and deep learning algorithms can effectively automate abstract screening tasks, saving valuable time and effort in the SLR process.

Although machine learning and deep learning algorithms trained on the two SLR corpora showed some variations in performance, there were also some consistencies. Firstly, adding additional citation features significantly improved the performance of conventional machine learning algorithms, although the improvement was not as strong in transformer-based deep learning models. This may be because transformer models were mostly pre-trained on abstracts, which do not include additional citation information like MeSH terms, keywords, and journal names. Secondly, when using only title and abstract as input, transformer models consistently outperformed conventional machine learning algorithms, highlighting the strength of subject domain-specific pre-trained language models. When all citation features were combined as input, conventional machine learning algorithms showed comparable performance to deep learning models. Given the much lower computation costs and faster training and prediction time, XGBoost or support vector machines with all citation features could be an excellent choice for developing an abstract screening system.

Some limitations remain for this study. Although we’ve evaluated cutting-edge machine learning and deep learning algorithms on two SLR corpora, we did not conduct much task-specific customization to the learning algorithms, including task-specific feature engineering and rule-based post-processing, which could offer additional benefits to the performance. As the focus of this study is to provide generalizable strategies for employing machine learning to abstract screening tasks, we leave the task-specific customization to future improvement. The corpora we evaluated in this study mainly focus on health economics and outcome research, the generalizability of learning algorithms to another domain will benefit from formal examination.

Extensive studies have shown the superiority of transformer-based deep learning models for many NLP tasks [11, 13–16]. Based on our experiments, however, adding features to the pre-trained language models that have not seen these features before may not significantly boost their performance. It would be interesting to find a better way of encoding additional features to these pre-trained language models to maximize their performance. In addition, transfer learning has proven to be an effective technique to improve the performance on a target task by leveraging annotation data from a source task [17–19]. Thus, for a new SLR abstract screening task, it would be worthwhile to investigate the use of transfer learning by adapting our (publicly available) corpora to the new target task.

When labeled data is available, supervised machine learning algorithms can be very effective and efficient for article screening. However, as there is increasing need for explainability and transparency in NLP-assisted SLR workflow, supervised machine learning algorithms are facing challenges in explaining why certain papers fail to fulfill the criteria. The recent advances in large language models (LLMs), such as ChatGPT [20] and Gemini [21], show remarkable performance on NLP tasks and good potentials in explainablity. Although there are some concerns on the bias and hallucinations that LLMs could bring, it would be worthwhile to evaluate further how LLMs could be applied to SLR tasks and understand the performance of using LLMs to take free-text article screening criteria as the input and provide explainanation for article screening decisions.

## Data Availability

The annotated corpora underlying this article are available at https://github.com/Merck/NLP-SLR-corpora.

## References

[1] Bullers K, Howard AM, Hanson A (2018). It takes longer than you think: librarian time spent on systematic review tasks. J Med Libr Assoc.

[2] Carver JC, Hassler E, Hernandes E et al. Identifying Barriers to the Systematic Literature Review Process. In: *2013 ACM / IEEE International Symposium on Empirical Software Engineering and Measurement*. 2013. 203–12. 10.1109/ESEM.2013.28.

[3] Lame G (2019). Systematic literature reviews: an introduction. Proc Des Society: Int Conf Eng Des.

[4] Michelson M, Reuter K (2019). The significant cost of systematic reviews and meta-analyses: a call for greater involvement of machine learning to assess the promise of clinical trials. Contemp Clin Trials Commun.

[5] Recent advances in. biomedical literature mining | Briefings in Bioinformatics | Oxford Academic. https://academic.oup.com/bib/article/22/3/bbaa057/5838460?login=true (accessed 30 May 2022). 10.1093/bib/bbaa057 PMC8138828 32422651

[6] Medical Subject Headings - Home Page. https://www.nlm.nih.gov/mesh/meshhome.html (accessed 30 May 2022).

[7] Chen T, Guestrin C, XGBoost:. A Scalable Tree Boosting System. In: *Proceedings of the 22nd ACM SIGKDD International Conference on Knowledge Discovery and Data Mining*. New York, NY, USA: Association for Computing Machinery 2016. 785–94. 10.1145/2939672.2939785.

[8] Noble WS (2006). What is a support vector machine?. Nat Biotechnol.

[9] *Logistic Regression*. 10.1007/978-1-4419-1742-3 (accessed 30 May 2022).

[10] Random forest classifier for remote sensing classification. International Journal of Remote Sensing: Vol 26, No 1. https://www.tandfonline.com/doi/abs/10.1080/01431160412331269698 (accessed 30 May 2022).

[11] Devlin J, Chang M-W, Lee K (2019). BERT: pre-training of Deep Bidirectional transformers for Language understanding. arXiv.

[12] Vaswani A, Shazeer N, Parmar N et al. Attention is All you Need. In: *Advances in Neural Information Processing Systems*. Curran Associates, Inc. 2017. https://proceedings.neurips.cc/paper/2017/hash/3f5ee243547dee91fbd053c1c4a845aa-Abstract.html (accessed 30 May 2022).

[13] BioBERT. a pre-trained biomedical language representation model for biomedical text mining | Bioinformatics | Oxford Academic. https://academic.oup.com/bioinformatics/article/36/4/1234/5566506 (accessed 3 Jun 2020). 10.1093/bioinformatics/btz682 PMC7703786 31501885

[14] Gu Y, Tinn R, Cheng H (2021). Domain-specific Language Model Pretraining for Biomedical Natural Language Processing. ACM Trans Comput Healthc.

[15] Chen Q, Du J, Allot A (2022). LitMC-BERT: transformer-based multi-label classification of biomedical literature with an application on COVID-19 literature curation. arXiv.

[16] Chen Q, Allot A, Leaman R (2022). Multi-label classification for biomedical literature: an overview of the BioCreative VII LitCovid Track for COVID-19 literature topic annotations. arXiv.

[17] Kermany DS, Goldbaum M, Cai W (2018). Identifying Medical diagnoses and Treatable diseases by Image-based deep learning. Cell.

[18] Howard J, Ruder S (2018). Universal Language Model fine-tuning for text classification. arXiv.

[19] Do CB, Ng AY. Transfer learning for text classification. In: *Advances in Neural Information Processing Systems*. MIT Press. 2005. https://proceedings.neurips.cc/paper/2005/hash/bf2fb7d1825a1df3ca308ad0bf48591e-Abstract.html (accessed 30 May 2022).

[20] Achiam J et al. Gpt-4 technical report. arXiv preprint arXiv:2303.08774 (2023).

[21] https://gemini.google.com/app/a4dcd2e2d7672354. (accessed 01 Feb 2024).

